# Electronic contribution in heat transfer at metal-semiconductor and metal silicide-semiconductor interfaces

**DOI:** 10.1038/s41598-018-29505-4

**Published:** 2018-07-27

**Authors:** Georges Hamaoui, Nicolas Horny, Zilong Hua, Tianqi Zhu, Jean-François Robillard, Austin Fleming, Heng Ban, Mihai Chirtoc

**Affiliations:** 1 GRESPI, Multiscale Thermophysics Lab., Université de Reims Champagne-Ardenne URCA, Reims, France; 2 Mechanical Engineering and Materials Science department, University of Pittsburgh, Pittsburgh, PA United States; 3 Univ. Lille, CNRS, Centrale Lille, ISEN, Univ. Valenciennes, UMR 8520 - IEMN, F-59000 Lille, France

## Abstract

This work presents a direct measurement of the Kapitza thermal boundary resistance *R*_*th*_, between platinum-silicon and platinum silicide-silicon interfaces. Experimental measurements were made using a frequency domain photothermal radiometry set up at room temperature. The studied samples consist of ≈50 nm of platinum and ≈110 nm of platinum silicide on silicon substrates with different doping levels. The substrate thermal diffusivity was found via a hybrid frequency/spatial domain thermoreflectance set up. The films and the interfaces between the two layers were characterized using scanning electron microscopy, transmission electron microscopy and energy-dispersive X-ray spectroscopy. X-ray diffraction was also used to determine the atomic and molecular structures of the samples. The results display an effect of the annealing process on the Kapitza resistance and on the thermal diffusivities of the coatings, related to material and interface changes. The influence of the substrate doping levels on the Kapitza resistance is studied to check the correlation between the Schottky barrier and the interfacial heat conduction. It is suggested that the presence of charge carriers in silicon may create new channels for heat conduction at the interface, with an efficiency depending on the difference between the metal’s and substrate’s work functions.

## Introduction

Considering the current rate of technology advancement, the race to find new materials with enhanced electrical/thermal properties is at its peak. With a low electronic resistivity (i.e. high electrical conductivity), metal silicides are considered as excellent conductors that form at low temperature, which renders them extremely useful in CMOS technologies (Complementary metal oxide semiconductor). For that reason, recent studies aim to understand the thermal conduction at the interfaces of these structures and measure their thermophysical properties. Due to their high electrical conductivity, they are used as ohmic contacts and as Schottky barrier diodes in silicon integrated circuits^1–4^. The silicides are normally formed from a Metal-Silicon couple (MS couple). The silicidation process, i.e. diffusion of the coating atoms into the substrate and vice versa, can occur using more than half of the elements of the periodic table.

One of the most interesting properties of MS interfaces is the Schottky Barrier Height (SBH). Its amplitude *ɸ*_*B*_, reflects the energy mismatch of the band edges between the charge carriers in the semiconductor and the Fermi level of the metal. Thus, the SBH defines the specific electrical contact resistivity at the interface of a MS. In metal-oxide-semiconductor field-effect-transistors (MOSFETs) for example, researchers seek materials with a low SBH^5^. Platinum monosilicide, PtSi, is a good candidate for this kind of applications for its thermal stability^6^ and has one of the lowest SBH of 0.22 eV with a p-type Si contact^7,8^. When coupled with n-type Si, PtSi exhibits a higher SBH of 0.8 eV^9,10^.

Nanoscale layers are generally used in micro- and nano- electronics, where the thermal transport is dominated by interfacial properties^11,12^. This interfacial thermal transport is characterized by the thermal boundary resistance (TBR) *R*_*th*_, known as the Kapitza thermal resistance, the inverse of the thermal boundary conductance *G*^13–15^. To our knowledge, there are few studies on the thermophysical proprieties and on the interfacial thermal transport for metal silicide materials. A recent study by Ye *et al*.^16^ revealed that the Kapitza thermal conductance at metal silicide-silicon interfaces is usually higher than the one at metal-silicon interfaces. This opens an opportunity to study new interfacial thermal transport phenomena, and to understand the fundamental heat transfer channels at these interfaces.

The specific objective of this study is to correlate the interfacial heat transport between metal and semiconductor with the metal’s electronic structure by measuring the Kapitza thermal resistance. The thermal boundary resistance and the silicon thermal diffusivity of these Pt-Si and PtSi-Si layered samples are found using frequency domain photothermal radiometry (FD-PTR)^17,18^ and hybrid frequency/spatial domain thermoreflectance (FSDTR)^19–21^ setups. For simplicity, we used PTR, FSDTR and TBR are used as abbreviations for the experimental methods and for the Kapitza thermal boundary resistance. The results contribute to improve the understanding of thermal transport at metal silicide-dielectric interfaces. Which encourages and enables better usage of these metal silicides in silicon-based devices.

## Results

### Sample characterisation

The process of creating metal silicides is called silicidation and it corresponds to the annealing of metal-silicon films. For example, after annealing, Ni-Si, Ti-Si and Pt-Si will transform to NiSi-Si^22^, TiSi_2_-Si^23^ and PtSi-Si^24,25^ respectively. By this silicidation process, the electrical and thermophysical properties of films and interfaces are modified. For that reason, this study aims to understanding the changes of the thermophysical properties. Platinum thin films were chosen due to their low and high SBH when in contact with p- and n-type silicon substrates, respectively.

During this work, ten experimental samples have been studied. Half of them consist of 55 nm thick platinum coatings (five samples) and the others of 110 nm thick platinum mono-silicide coatings (five samples) on 400 µm thick silicon substrates with different doping levels. Five Pt thin layers were coated by electron beam evaporation (EB-PVD) on a (100) silicon bulk substrate. Then, a portion of each sample was annealed in a chamber filled with N_2_H_2_. During three minutes the temperature is ramped up to 400 °C and then kept constant for 2 additional minutes enabling a complete reaction of the platinum layer according to Larrieu *et al*. and Breil *et al*.^26,27^. The Si doping level goes from n+ type to p+ type region. The different dopant concentrations of Si substrate are deduced from the resistivity given by the manufacturer and are shown in Table 1.

**Table 1:** Table grouping the doping concentration and the thickness of the ten different samples (five unannealed forms of Pt on Si substrates with different doping levels, and five annealed forms of Pt on the same Si substrates).

Samples	Si doping	Resistivity (Ω cm)	Doping concentration (atoms cm^−3^)	Thickness of the unannealed Pt (nm) ± 10%	Thickness of the annealed PtSi (nm) ± 10%
S1	n+	0.03	7,2E + 17	58.1	90.5
S2	n	10	4,5E + 14	49.1	111
S3	Intrinsic	—	—	56.7	111
S4	p	7.5	1,8E + 15	55.8	105
S5	p+	0.06	6,8E + 17	55.8	116

**Figure 1:**
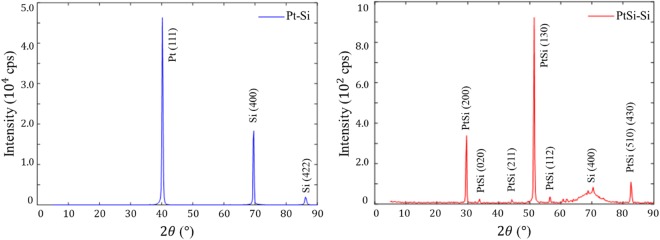
X-ray diffraction (XRD) patterns for unannealed Pt-Si samples (in blue) and annealed PtSi-Si samples (in red).

X-ray diffraction (XRD) patterns and energy-dispersive X-ray (EDX) images were made to study the layers structure, and to check the transformation of the platinum films. The XRD patterns for S1 samples are grouped in Fig. 1 and the EDX results are detailed in the supporting information (Supplementary Figs S1–S2 and Table S1).

The XRD patterns for both types of samples are in good agreement with other studies^16,28^. Pt and Si crystalline peaks are clearly present for the unannealed samples. After annealing, all the Pt was consumed and transformed to PtSi (red plot in Fig. 1). This was also confirmed by the EDX, where the presence of Pt for the unannealed samples was found equal to 100% at 20 nm of the interface. As for the annealed samples, an atomic ratio of 50% for Pt and 50% for the Si inside the PtSi layer was established. These results confirm the transformation of the platinum into the platinum monosilicide.

**Figure 2:**
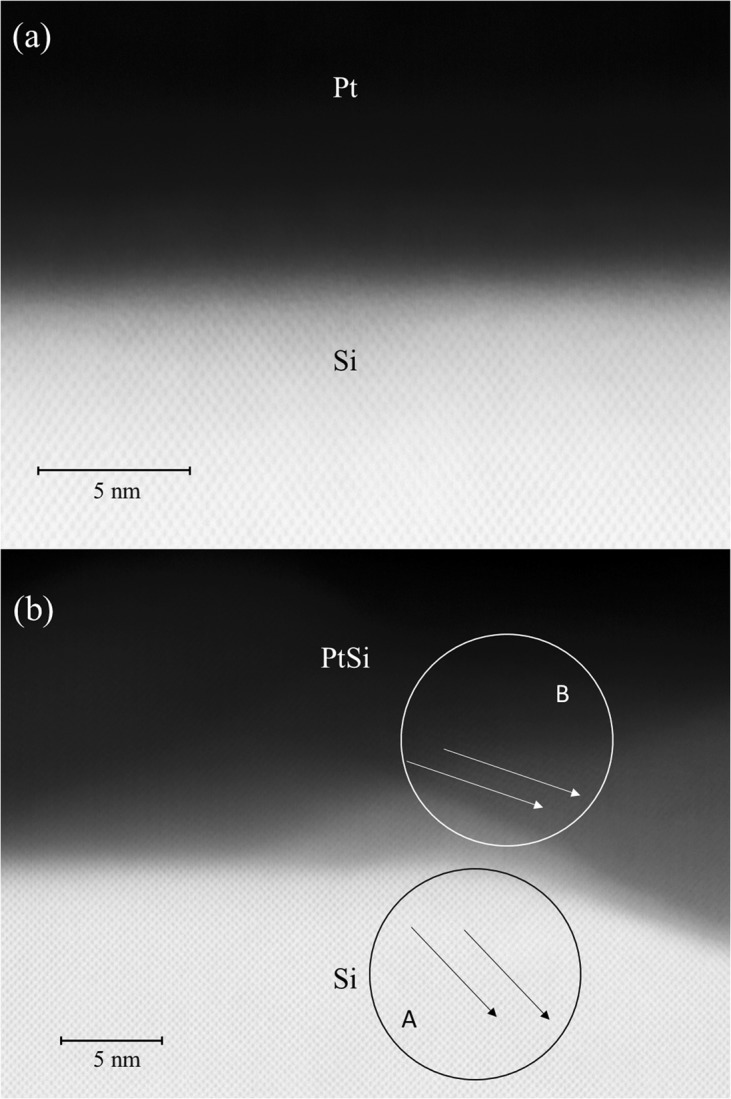
Example of a TEM image of the: (**a**) unannealed S3 and (**b**) annealed S3 samples.

PtSi molecule has an orthorhombic, B31 type of structure^29^. The lattice parameter of this monosilicide is 10% smaller than that of silicon which causes a lattice mismatch between the PtSi and the (100) Si substrate^30^. Due to this difference of lattice parameters many Moiré fringes patterns (grains) start appearing at the interface for temperatures around 250 °C. But when the samples were heated between 350 and 800 °C, larger grains were observed at the interface^31,32^. To check the effect of the annealing at 400 °C, a transmission electron microscopy (TEM) was used on the present samples (Fig. 2 groups the results for the unannealed and annealed S3 sample’s interfaces). Figure 2a shows that there is no intermediate layer between the Pt and the Si after deposition. On Fig. 2b, it seems that after annealing at 400 °C the structure of the grains is not big enough to disturb the continuity of the film, i.e., there is a low roughness at the interface and the continuity of the film is preserved. On the other hand, in both figures, the diamond structure of the Si lattice is clearly present. For unannealed sample (Fig. 2a) the diamond structure stops at the interface, whereas for the annealed one (Fig. 2b), the Moiré fringes are joining at the interface, which proves that there is also no layer between PtSi and Si. However, the fringes of the silicon and the silicide do not have the same direction (Zone A and B in Fig. 2b), which means that the PtSi layer is not epitaxial on the Si(100).

The stability of the PtSi grown on a (100) Si can change with the increase of temperature. For temperatures higher than 100 °C, it was found that the epitaxial PtSi film on (111) Si is more stable than the nonepitaxial film on (100) Si^33^. Even if this is the case, a recent study on PtSi-Si interfaces determined that there is no dependence of TBR on the epitaxy of the film^16^. For comparison, the heating induced by the PTR and the FSDTR methods is smaller than 10 °C, and all measurements were carried out at room temperature.

### Thermophysical proprieties

To find the TBR using the photothermal techniques, the thermal conductivities *k*, the volumetric heat capacities *C*_*v*_ of both layers (product of specific heat capacity *C*_*p*_ and density *ρ*), and the coating film thickness *l*_*film*_ must be known. For that reason, sensitivity calculations^34,35^ were performed for both experimental methods on the most influential fitting parameters. FSDTR is found to be sensitive to the substrate thermal diffusivity since in the operating frequency range, the cross-plane thermal diffusion length $\mu ={(a/\pi f)}^{1/2}$ is larger compared to the layer thickness. Whereas, by using a higher frequency range, the PTR is more sensitive to the TBR (see Supplementary information Fig. S3). Thus, the two methods were used jointly to characterize the ten samples by finding both the substrate’s thermal diffusivities and the TBRs. Subsequently, the calculated sensitivities were integrated in a least squares algorithm^36,37^ to calculate the total uncertainty on the fitting parameters by considering both the experimental noise and the supposed known parameter’s uncertainties. This uncertainty calculation was used for both experimental methods and the details are presented in the supplementary information (Table S2).

**Figure 3:**
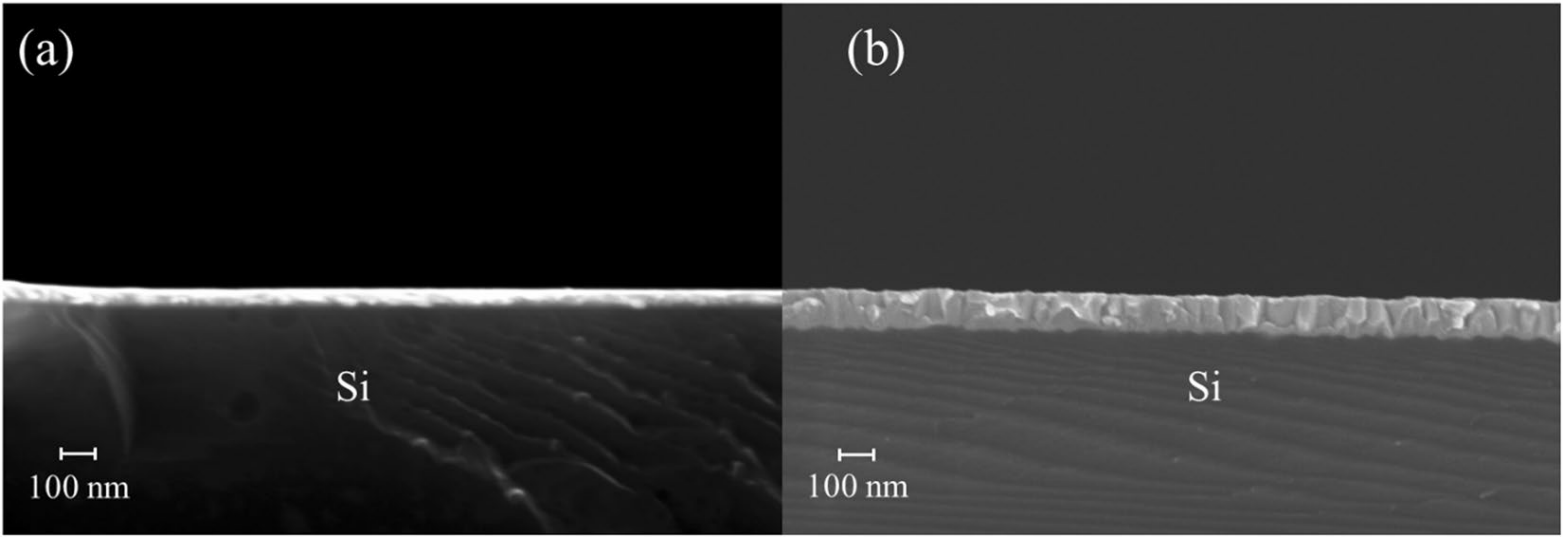
Example of a SEM images for the S3 samples: (**a**) unannealed Pt-Si and (**b**) annealed PtSi-Si samples.

Due to low experimental sensitivity to the films properties, their *k* and *C*_*v*_ were taken from literature studies (by including their respective uncertainties in the least squares algorithm). The film thicknesses were measured by a scanning electron microscope (SEM) and an example is presented in Fig. 3. The measured thicknesses are in good agreement with other studies which suggest that after the silicidation, the thickness of the new PtSi layer is about 200% the original Pt layer^26,31^. The values are grouped in Table 1 with an uncertainty of 10% (error using the SEM measurements).

**Figure 4:**
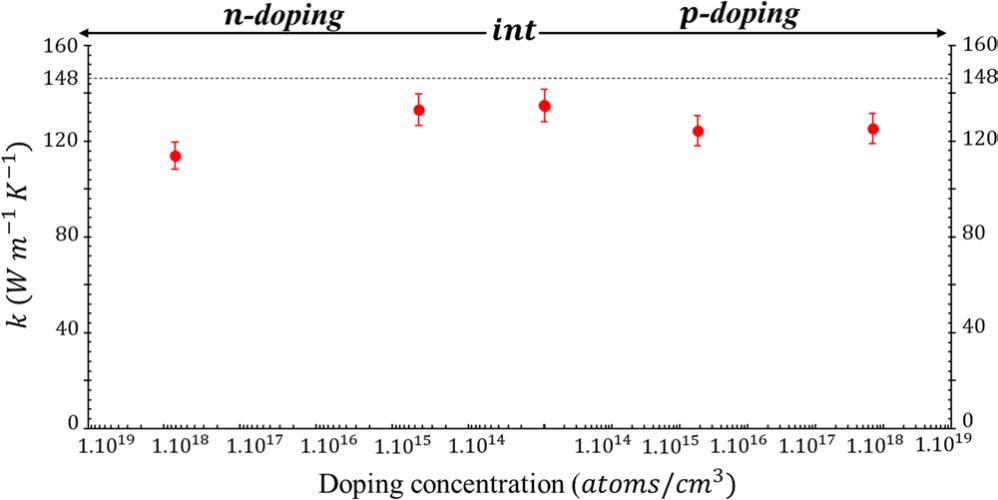
FSDTR results for the averaged thermal conductivity of the silicon substrate for both annealed and unannealed samples, with a dashed line as a reference of the thermal conductivity of the bulk Si equal to 148 *Wm*^−1^*K*^−1^.

The FSDTR measurements give the thermal conductivities of the Si substrates while their *ρ* and *C*_*p*_, were considered the same as for the bulk^38^. A small difference between the two substrates’ *k* (annealed and unannealed samples) was found, probably due to poor signal-to-noise ratio for unannealed samples. An average *k* of both types of substrates was used for the calculations performed for Fig. 4. These properties are in good agreement with other literature studies. The *k* of the substrate decreases with the doping level due to phonon scattering by the impurities^39^.

Both *ρ*, and *C*_*p*_, of Pt films were considered the same as for bulk Pt values and were taken from literature^38^. Concerning its *k*, it was demonstrated that it changes with the thickness of the layer due to limitation of phonon mean free path^11,40^. Studies on platinum nanofilms showed that its *k* is lower than in the bulk. Specifically, *k* of the film is less than half of the bulk value, around 30 *W*m^−1^*K*^−1^ for thicknesses around 50 nm^41,42^. To our knowledge, there are few direct experimental calculations of PtSi $k$. Ye *et al*.^16^ found an estimation of 18 *W*m^−1^*K*^−1^ using the Wiedemann-Franz (WF) law after electrical resistivity measurements. The presence of PtSi molecules instead of Pt atoms may disrupt the phonon coupling inside the layer even if its thickness is doubled. This may cause the reduction of the thermal conductivity of the PtSi film compared to the Pt one. Additionally, for the PtSi layer, *C*_*v*_ was calculated using a density functional theory (DFT) in the work of Ye *et al*.^16^. They found *C*_*v*_ = 2.49 10^6^ *J*m^−3^*K*^−1^ at room temperature. The density of the PtSi was reported as 12 378 *kg* m^−3^ by Schubert and Pfisterer^43^.

**Figure 5:**
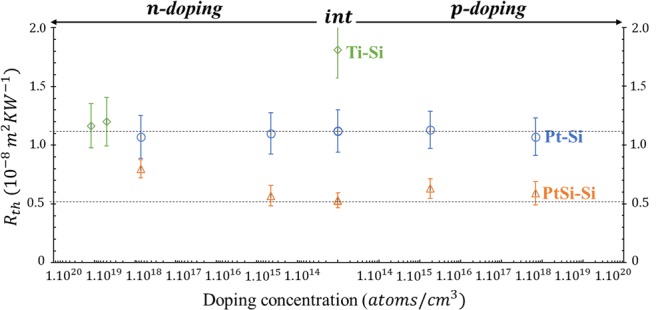
TBR results for Pt-Si and PtSi-Si samples using the PTR setup. Additionally, Ti-Si TBR (with small adjustments) from a previous study^44^ is shown. The two dashed lines for both S3 unannealed and annealed TBRs are used as guides for the eye.

Using the previous values, an estimation of the TBR can be given by the fitting algorithm. These results are shown in Fig. 5. From this figure, the annealed samples have a lower TBR than the unannealed samples despite showing lower film thermophysical properties (as explained in the previous paragraph). The interdiffusion of atoms at the interface may cause the improved heat transfer from the PtSi layer to the Si by creating new bonds between the two layers. Additionally, there is also a slight influence of the substrate doping level on the TBR results within the errors. I.e., with the increase of the Si doping level, a small decrease of Pt-Si TBR and a slight increase of PtSi-Si TBR are observed, which are mainly correlated to the substrates thermal conductivities changes (Fig. 4). These results are in good agreement with the study of Ye *et al*., and the values found here are very close to the ones already found by time domain thermoreflectance (TDTR) at higher frequency ranges^16^.

Furthermore, the results of a previous study on Ti-Si contact^44^ is added to Fig. 5 for comparison (absolute values of TBR are adjusted compared to ref.^44^ due to the changes of the thermal conductivity of Ti thin layer and Si substrate). In this case, the variation of TBR is significant when the doping level increases.

## Discussion

**Figure 6:**
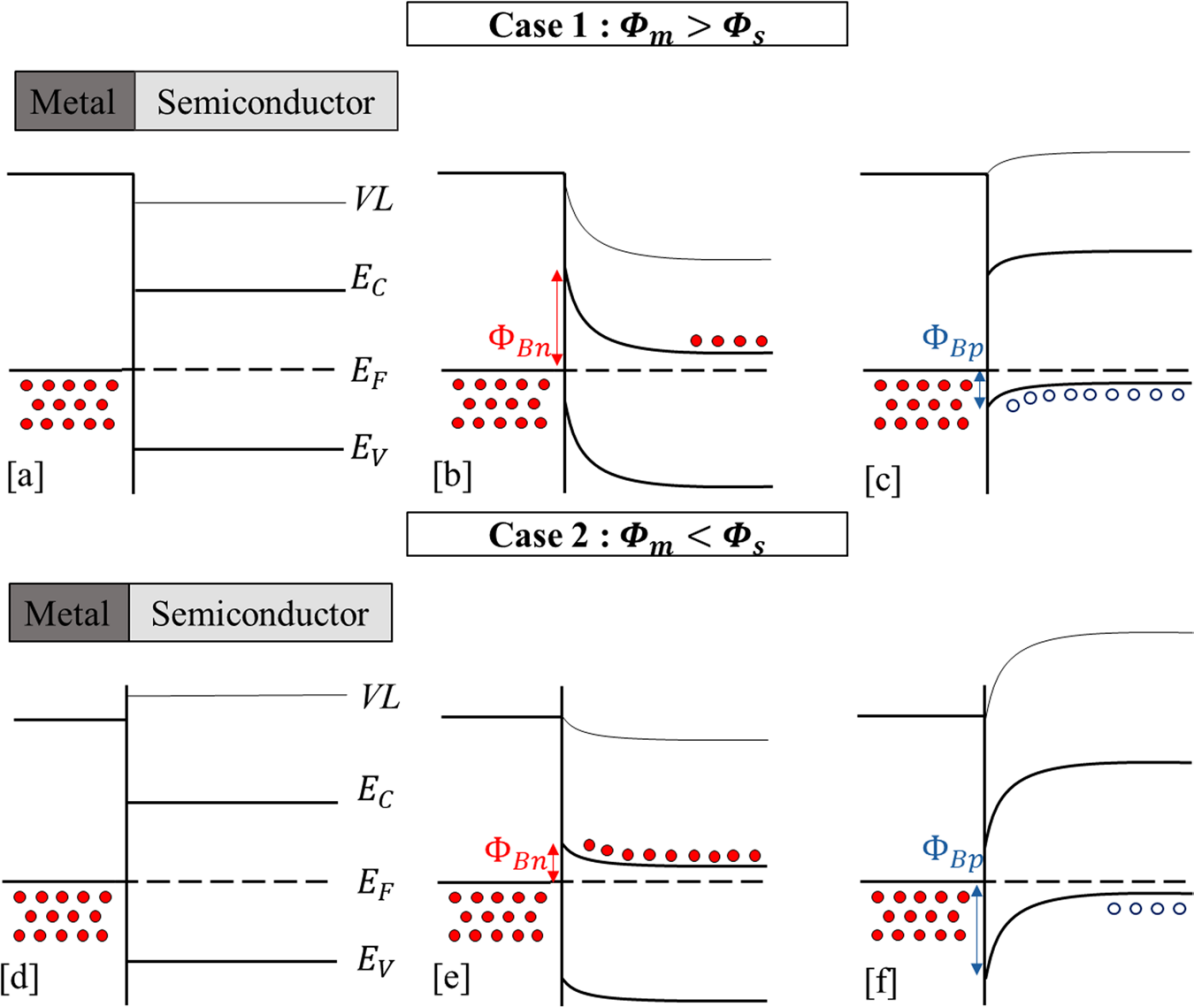
Energy diagrams for both types of MS interface, when Φ_*m*_ > Φ_*s*_ (Case 1) and when Φ_*m*_ < Φ_*s*_ (Case 2). With: intrinsic semiconductor (**a**,**d**), n-type semiconductor (**b**,**e**), p-type semiconductor (**c**,**f**); *ɸ*_*Bp*_ and *ɸ*_*Bn*_ represent respectively the SBH for holes and for electrons; VL is the vacuum level; *E*_*f*_, *E*_*c*_ and *E*_*v*_ are the Fermi level, the conduction band energy and the valence band energy respectively; the red dots are the electrons in the conduction band, and the blue circles are the holes in the valence band.

For MS couples, the interfacial heat transfer is led mainly by phonons, i.e. conduction through the substrate, for temperatures higher than 150 K. It is due to the coupling between the phonons of the metal and the phonons of the semiconductor. However, many studies demonstrate the importance of considering not just the elastic phonon scattering but also inelastic phonons and the electron-phonon interfacial coupling between the metal and the non-metal interfaces^45–47^. These effects of electron-phonon couplings are caused by the thermally excited leakage carriers over the Schottky barrier between the two layers as explained by Oto *et al*.^31^. This phenomena changes depending on the type of MS couple, and there are generally two cases: when the work function (${\rm{\Phi }}(eV)$) of the metal is smaller than that of the semiconductor, or the opposite^30,48^. Figure 6 describes the two cases.

These energy bands explain that the MS couples differs from a low to high Schottky barriers to holes (*ɸ*_*Bp*_) and to electrons (*ɸ*_*Bn*_) used with p- and n-type doping silicon structure. What is interesting is that the SBH between a metal and a n-type Si can be lowered through an image-force mechanism, by increasing the n-doping^49,50^. This means that the presence of electrons near the interface will cause the Schottky barrier to be narrower and this effect changes with the doping level. For low doping, the electrons mainly go through the barrier via thermionic emission. Intermediate doping adds the field emission (tunnelling) effect to the thermionic effect. For high doping levels, the tunnelling will be the dominating channel for the electrons to cross the barrier.

With a n- and p-type semiconductor in case 1 (when the metal work function Φ_*m*_ is larger than the semiconductor work function Φ_*s*_), the contacts are respectively a Schottky contact (Fig. 6b) and an ohmic contact (Fig. 6c). In case 2, when Φ_*m*_ is smaller than Φ_*s*_, with a n-type semiconductor the contact is an ohmic contact (Fig. 6e), and with a p-type dielectric it is a Schottky contact (Fig. 6f)^30,48^.

Experimentally, case 1 represents Pt-Si and PtSi-Si contacts (with *ɸ*_*Bp*_ = 0.2  eV^7,8^ and *ɸ*_*Bn*_ = 0.8 eV^9,10^ for PtSi-Si taken from literature). This is since the Pt and the PtSi have a work function of 5.7 eV and 5.02 eV respectively^7^, which are higher than the Si work function (4.9 eV with a p-doping, and 4.7 eV for a n-doping)^51,52^. Thus, the TBR’s behaviours at the metal-silicon interfaces (Fig. 5) can be explained by the energy bands of Fig. 6.

The contact between Pt or PtSi and the n-type Si (sample S1) is a Schottky contact. When the metallic side is heated by the laser, the electrons can cross the barrier *via* thermionic emission. But due to a space charge zone, the electrons are far from the interface, which renders the electronic coupling difficult (coupling between electrons of the metal and electrons of the semiconductor). For the p-doped Si (sample S5) the contact is ohmic, but the electric conduction is made by holes having lower energy than the Fermi level. After the laser heating, the “hot” electrons in the metal (having an energy above the Fermi level) cannot couple directly with the holes from the valence band of the semiconductor. Therefore, there is no thermal energy transport by electron-phonon coupling within the crystalline matrix. For this type of contact (Pt-Si or PtSi-Si couples) the effect of the barrier on the heat transfer is very small, and this is clearly seen by the results in Fig. 5 (bearing in mind the uncertainties). Moreover, to check the consistency of this theory (correlation between energy bands and TBR), we included in Fig. 5 a result from a former study on Ti/n-Si interfaces^44^. There it was found that the TBR of Ti-Si changes with the n-doping level of Si, and as one can see, the changes are significant. Having a work function equal to 4.06 eV^52^, Ti-Si couple is close to case 2 (in Fig. 6), and with a highly doped n-type Si (higher than 10^19^ atoms/cm^3^) the contact is ohmic (with *ɸ*_*Bp*_ = 0.6 eV and *ɸ*_*Bn*_ = 0.5 eV)^53^. Since there is no space charge zone, the “hot” electrons can then enhance the effect of the thermal coupling by electrons through the interface, resulting in large TBR decrease. For this case, we suggest the presence of a significant electron-electron coupling at the interface which sets in as an additional channel to the phonon-phonon and electron-phonon channels. But its influence is smaller compared to the other two ones, as mentioned in previous studies^44,54^.

These results lay down a new way to study and to use metal silicide-silicon couples. Upcoming research is in progress to study the TBR at the interface of diverse set of metal- and metal silicide- silicon couples, like Ti or Er due to their different work function and SBH with Si substrates. Also, the influence of an electric potential (with direct or inverse polarization) on the heat transfer at the interfaces of these MS couples is envisaged. We think it is possible that for some MS couples an electrical polarization can induce an enhancement of the interfacial heat conduction due to the electronic coupling. These effects will be considered as a Schottky thermal diode (with a heat rectification effect) or even as an ohmic thermal contact (where the heat can be efficiently transmitted both ways). Additionally, we are working on a modified AMM/DMM model which considers the electron-electron and the electron-phonon interfacial couplings to verify the correlation of the SBH and the TBR.

## Conclusion

In this article, we reported the measurements of the Kapitza resistance at metal-silicon and metal silicide-silicon interfaces. The thermal boundary resistance (TBR) for the annealed samples is found lower than that for the unannealed one, meaning that the heat transfer from metal to silicon is improved due to new bonds created by the interdiffusion of the two layers. For platinum and platinum monosilicide samples, a constant TBR is found for different doping concentrations, which may be attributed to the presence of a charge carrier’s distribution far from the interface and to the Schottky barrier (energy bands case 1 in Fig. 6). Via a comparison to a previous study on titanium and n-type silicon couples, the change of TBR values is ascribed to the presence of a “hot” electrons current at the interface (balanced by a “cold” electron current in the opposite semiconductor-metal direction). For that reason, we suspect the existence of a new heat transfer mode at the interface, which should be related to the coupling of electrons from the metal and electrons from the n-doped semiconductor. The barrier modifications can increase or decrease the electron-phonon and electron-electron couplings between the metal and the dielectric with n- and p-doping. This new hypothesis deserves to be systematically investigated since its implications are critical for theoretical models. Such mechanisms can improve the heat transmission at the interface of silicon-based devices and open novel ways to use them, like in Schottky thermal diodes or even in thermal ohmic contacts.

## Methods

### Sample preparation

The annealing of platinum over a silicon substrate can generate three types of compositions: PtSi, Pt_2_Si and Pt_3_Si^55,56^. Only the first two forms are stable at room temperature (RT)^24^. The first step of the PtSi fabrication is the diffusion of Pt into Si to form the Pt_2_Si. After the complete consumption of the film, the second reaction will begin, Si diffuses back into the newly formed Pt_2_Si to form the final layer of PtSi^24,25^. Each reaction has a specific activation energy. The formation of Pt_2_Si takes place at temperatures between 245–258 ^o^C and the formation of PtSi happens for temperatures of 324–338 °C^26,57^. These two reactions are very fast, and the complete silicidation occurs in two minutes^26^. In our case, Pt thin layers were coated by electron beam evaporation (EB-PVD) on a (100) Si bulk substrate. The deposition is made in ultra-vacuum using a Plassys Meb 550 s system. The samples were cleaned using an Argon source inside the inner chamber of the EB-PVD before the deposition. The deposition reaction is complete in absence of oxygen, because it was found that in the presence of oxygen, the deposition process is perturbed and the morphology of the interface can change^24,32^. Then, the annealing process is made at 400 °C for 2 minutes as was reported by Larrieu *et al*.^26^. The annealing is completed using a Four Jipelec JetStar 100S with a heating rate of 2,22 °C/Sec.

### FSDTR measurements

This setup represents a classical thermoreflectance method. The heating of the sample is provided by a laser at 532 nm of wavelength (pump beam), modulated up to 500 KHz by an acousto-optical modulator (AOM) (AOM, NEOS technologies 23080 with driver model N21080-1SAS). The thermal response is retrieved using another laser at 655 nm of wavelength (probe beam). Its reflection from the sample surface is captured by a photodiode (New Focus nanosecond photo detector 1621) and the signal is processed by a lock-in amplifier (Stanford Research System SRS830 or SRS844). The details of this setup are explained in the work of Hua *et al*.^58,59^.

By using the FSDTR in the spatial domain, the thermal diffusivities *a*(*m*^2^*s*^−1^) (with *a *=* k*/*C*_*v*_) of Si substrates are determined. 3D spatial and frequency domains models are used to solve the heat equation and to find the thermophysical parameters for both film and substrate.

### PTR measurements

The PTR measurements are based on the study of the modulated black body IR radiation emitted by the samples after being optically heated. For that, a continuous laser at 532 nm of wavelength and modulated up to 10 MHz by an AOM (AOM model AA.MTT.AR 05) was used as heating source. The emitted IR signal is then collected by two parabolic off-Axis Au-coated mirrors (Edmund Optics) and transferred to a mercury cadmium telluride (MCT) (Kolmar Technologies, KMPV 11-1-J1/Ge) cooled detector. The detected signal is proportional to the variation of the sample temperature which depends on its thermophysical properties, via δ*V* = *p*_*c*_ *δT*. *V* is the voltage signal of the detector, *T* the temperature at the surface of the sample and *p*_*c*_ the proportionality coefficient. Physically, *p*_*c*_ is the product of the absorbed heat flux, the sample emissivity and the optical transfer function of the setup. The experimental setup used for this study is described elsewhere in more detail^44^. At high enough frequencies, the cross-plane thermal diffusion length in the sample $\mu ={(a/\pi f)}^{1/2}$ is smaller than the lateral size of the heating laser spot, which justifies a one-dimensional (1D) heat flow analysis for the alternative surface temperature *T*_*AC*_^44^. The previously derived equation serves as base to model the surface heating of a one-layer sample on top of a semi-infinite substrate. A Gauss-Newton (GN) minimization algorithm is then used with the TBR and the multiplicative factor *p*_*c*_ as fitting parameters.

### Sensitivity calculations

The relative sensitivity *S*_*p*_ of a function *F* is defined as the logarithmic derivative of *F* with respect to one of the parameters *p*: ${S}_{p}=\,\frac{p}{F}\frac{\partial F}{\partial p}$. The relative sensitivities are then found to be equal to ${S}_{p}^{A}=\frac{\partial \,\mathrm{ln}\,A}{\partial \,\mathrm{ln}\,p}$ and ${S}_{p}^{\phi }=\frac{\partial \phi }{\partial \,\mathrm{ln}\,p}$ ^34,35^ where ${S}_{p}^{A}$ and ${S}_{p}^{\phi }$ are the relative sensitivities related to the effect of parameter *p* variation on the amplitude and phase profiles respectively. Both sensitivities apply for the PTR measurements, and just the phase sensitivity for the FSDTR ones. An example using the unannealed intrinsic sample (S3) is displayed in Fig. S3 in the supplementary information.

### Uncertainties calculations

In this study the uncertainties were calculated via a least square algorithm (see supplementary information for more details). The formula considers both the experimental noise and the supposed known parameter’s uncertainties.

### Data availability

The datasets generated and/or analysed during the current study are available from the corresponding author on request.

## Electronic supplementary material

Supplemental information
