# Supplementary material for: Electronic contribution in heat transfer at metal-semiconductor and metal silicide-semiconductor interfaces
Source: Sci Rep. 2018 Jul 27;8:11352. doi: 10.1038/s41598-018-29505-4 (PMC6063978; doi:10.1038/s41598-018-29505-4)
Supplement: Supplementary file 1 — Supplemental information [file 41598_2018_29505_MOESM1_ESM.pdf]

Supplemental information for: “Electronic contribution in heat transfer at metal-semiconductor and metal silicide-semiconductor interfaces”

Georges Hamaoui[1], Nicolas Horny[1*], Zilong Hua[2], Tianqi Zhu[3], Jean-François Robillard[3], Austin Fleming[1,2], Heng Ban[2], Mihai Chirtoc[1]

[1]GRESPI, Multiscale Thermophysics Lab., Université de Reims Champagne-Ardenne URCA, Reims, France
[2]Mechanical Engineering and Materials Science department, University of Pittsburgh, Pittsburgh, PA, United States
[3]Univ. Lille, CNRS, Centrale Lille, ISEN, Univ. Valenciennes, UMR 8520 - IEMN, F-59000 Lille, France

[*]nicolas.horny@univ-reims.fr

This document contains additional information to the manuscript entitled **“Electronic contribution in heat transfer at metal-semiconductor and metal silicide-semiconductor interfaces”.** It provides supplementary details on the sample analysis, sensitivity and uncertainty calculations and on experimental data analysis in support of the manuscript.

1. Energy-dispersive X-ray spectroscopy (EDX) results:

EDX images were made to compare the atoms dispersion before and after annealing. The following results are EDX images for S3 unannealed and annealed samples, with an uncertainty of 10% on the measurements.

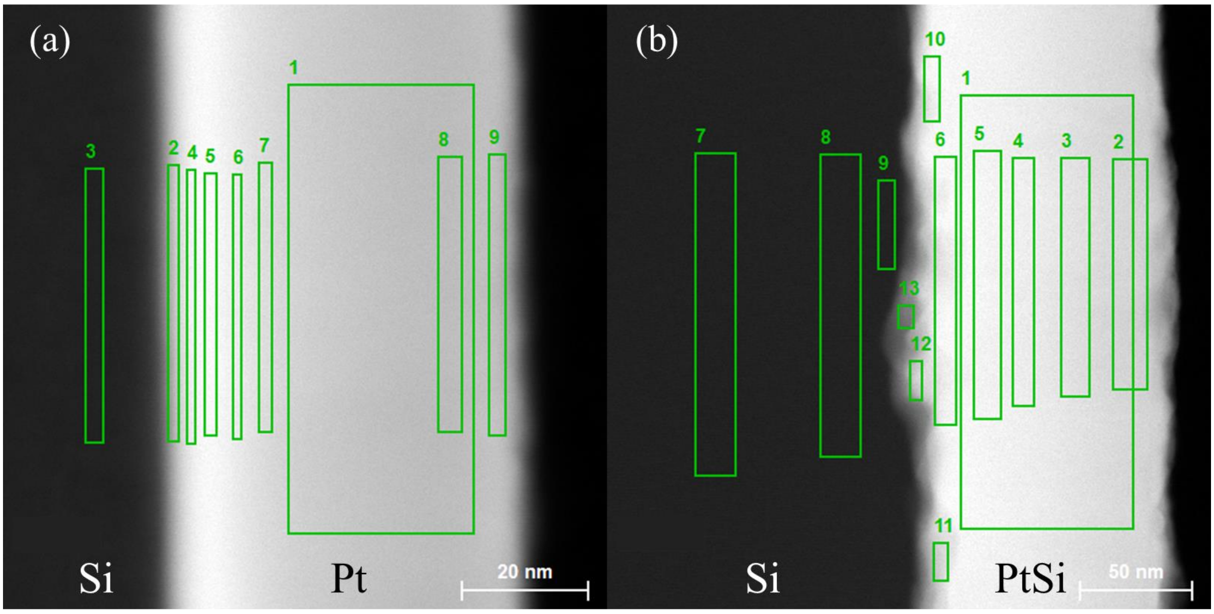

Figure S.1: EDX map images for: (a) unannealed S3 Pt-Si sample; (b) annealed S3 PtSi-Si sample.

Table S.1: EDX results for both types of samples knowing that the atomic number of Pt is 78 and that of Si is 14

Zones (Fig. S1)	Unannealed				Annealed			
	Normalized Weight %		Normalized Atomic %		Normalized Weight %		Normalized Atomic %	
	Si	Pt	Si	Pt	Si	Pt	Si	Pt
**1**	1.5	98.5	9.5	90.5	13	87	51	49
**2**	19	81	61.5	38.5	13	87	50.8	49.2
**3**	97.8	2.2	99.7	0.3	13	87	50	50
**4**	9.5	90.5	42	58	13	87	50.6	49.4
**5**	5	95	28	72	13	87	52	48
**6**	3	97	19	81	16	84	57	43
**7**	2	98	14.5	85.5	99.9	0.1	99.99	0.01
**8**	1.4	98.6	9	91	99.6	0.4	99.9	0.1
**9**	1	99	9	91	98.5	1.5	99.8	0.2
**10**					16.5	83.5	58	42
**11**					23	77	68	32
**12**					28	72	73	27
**13**					50	50	87.4	12.6

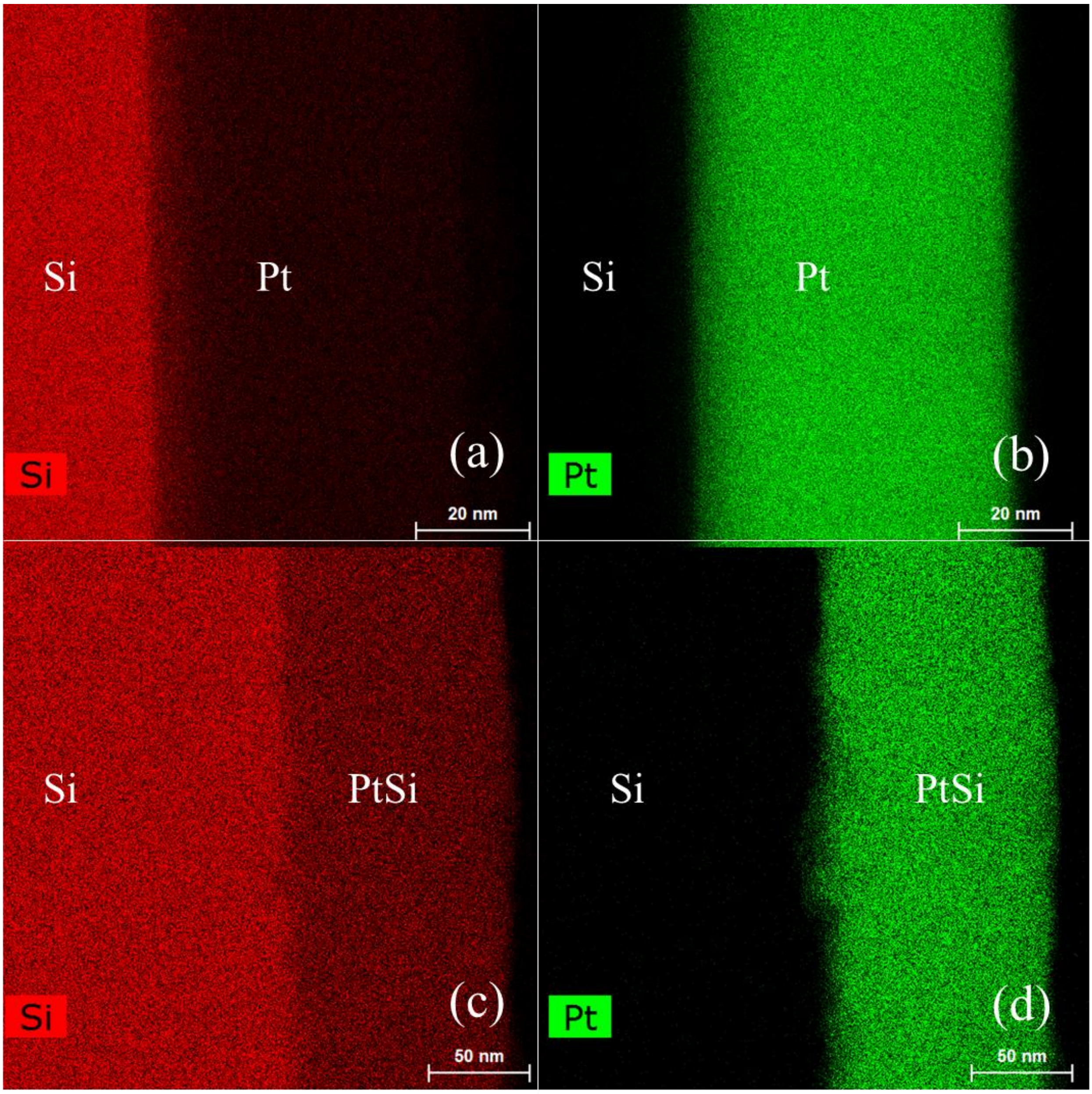

Figure S.2: EDX images for Si (in red) and Pt (in green) atoms for both types of samples: (a) and (b) for the unannealed S3 Pt-Si sample; (c) and (d) for the annealed S3 PtSi-Si sample.

2. Sensitivity and uncertainty calculations:

Both experimental methods use a mathematical minimization to obtain the selected fitting parameters. However, two types of minimization exist, one uses the amplitude and the phase of the thermal signal for the calculation, and the other one just the phase. When considering both amplitude and phase profiles, the minimization includes more experimental data in the analysis, leading to a better estimation of the fitting parameters. On the other hand, for a spatial scan on the sample surface, the local reflectivity may vary from one place to another, introducing additional errors in the FSDTR calculations. For that reason, in the paper, both amplitude and phase profiles are taken for the PTR minimization and just the phase profile for the FSDTR

one. Beforehand, sensitivity calculations[1,2] were performed for both experimental methods on the most influential fitting parameters. An example containing the intrinsic parameters of the unannealed sample S3 (thickness, substrate thermal properties…) is displayed in Fig. S.3.

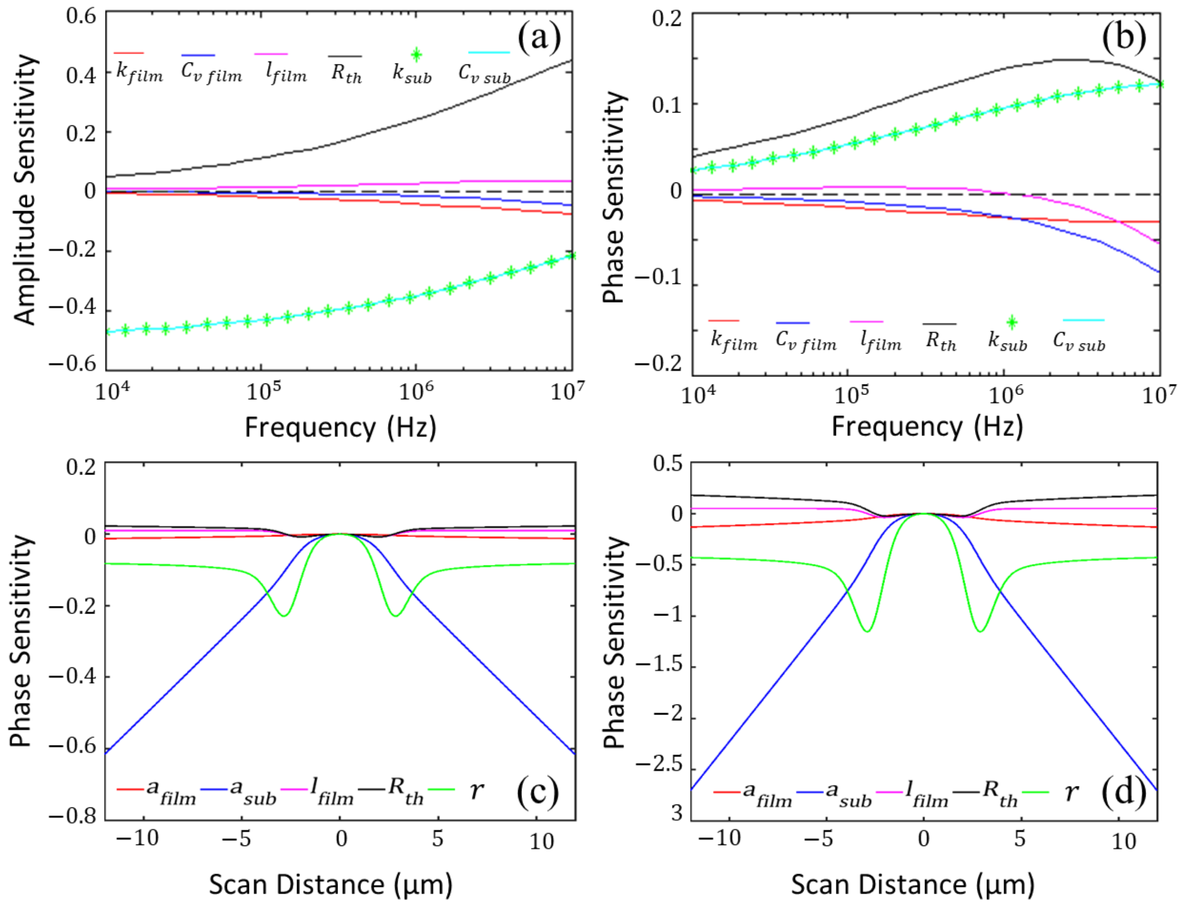

Figure S.3: PTR Amplitude (a) and phase (b) relative sensitivities of the surface temperature T_{AC} to the parameter p. FSDTR phase relative sensitivity for 10 KHz (c) and for 200 KHz (d). Subscripts film and sub represent respectively the coating film and the substrate. r and R_{th} are the radius of the probe laser beam and the TBR respectively.

Figures S.3(a) and (b) prove that the PTR experiment is sensitive to the TBR (solid black line) and to the properties of the substrate (green dots and blue line). Interestingly, in the used frequency range, these measurements are less sensitive to the thermal conductivity of the film. As for the FSDTR (Figures S.3(c) and (d)), the measurements are sensitive to the substrate diffusivity. For that reason, the thermal conductivities of the films were taken from other literature studies.

These sensitivities are then integrated into a least squares algorithm to calculate the total uncertainty on the fitting parameters[3,4]. Equations S.1 and S.2 express two types of errors. Where, $\sigma_{residual}$ and $\sigma_{\mathrm{supp}\,known}$ are respectively the uncertainties related to the variance of the noise and that of the supposed known parameters.

$$\sigma_{residual} = \sigma_{noise}[(X_r^t X_r)^{-1}]^{-1/2} \tag{S.1}$$

$$\sigma_{\text{supp}\,known} = -[(X_r^t X_r)^{-1} X_r^t X_c \, e_{\beta c}] \quad \text{(S.2)}$$

σ_{noise} is the residual noise between the model and the experimental data; X_r is the sensitivity matrix of the fitting parameters ($R_{th}$and p_c); X_r^t is the transpose of X_r; X_c is the sensitivity matrix of the parameter supposed known (thermophysical proprieties of the layers) and $e_{\beta c}$ the uncertainty vector of the supposed known parameters.

In order to combine these two uncertainties, a quadratic sum from the Guide to the Expression of Uncertainty in Measurement (GUM) [5] is used:

$$\Delta R_{th}^2 = 1.96^2 \left(\sigma^2{}_{residual} + \sigma^2{}_{\text{supp}\,known} \right) \quad \text{(S.3)}$$

The scaling factor 1.96 is given by the table of the Standard Normal Distribution for the repartition function on the detector opening for a confidence interval of 95%.

In the article the error bars are given by the least square algorithm using the errors on the supposed known parameters $e_{\beta c}$, taken from literature or from the SEM images[6–8]. The error percentages are grouped in Table S.2:

Table S.2: Error on the supposed known parameters taken from literature

$e_{\beta c}$ (%)	**Unannealed samples**	**Annealed samples**
k_{film}	10	10
$C_{v\,film}$	10	10
k_{sub}	5	5
$C_{v\,sub}$	5	5
l_{film}	5 nm	5 nm

3. PTR and FSDTR experimental results:

Using the FSDTR it was possible to find the thermal diffusivity of the Si substrate and the results were grouped in the following table. The density ρ and the specific heat C_p, are considered the same as the bulk values for Pt and Si.[6] For the PtSi layer, the volumetric heat capacity$C_v = 2.49\ 10^6\ J\ m^{-3} K^{-1}$ at RT was calculated using the density functional theory (DFT) in the work of Ye *et al.*[7] They did find. The density of the PtSi was reported as 12378 $kg\ m^{-3}$ by Schubert and Pfisterer in 1950.[8]

As presented in Table S.3, the thermal conductivity of the PtSi film is decreased compared to the Pt one, but the interfacial heat transfer is improved, as explained below. This can be noticed by comparing the Pt-Si and PtSi-Si phases. Figure S.4(a,b) presents the experimental amplitudes and phases of both samples types.

Table S.3: FSDTR results for the thermal diffusivity of the substrates and literature values for the thermal diffusivity, thermal conductivity and volumetric heat capacities of the films and substrates.

	$a\ (10^{-6}m^2s^{-1})$ $\pm$ 10%	$\rho\ (kg\ m^{-3})$ $\pm$10%	$C_p(J\ kg^{-1}\ K^{-1})$ $\pm$10%	$C_v(MJm^{-3}K^{-1})$ $\pm$10%	$k\ (W\ m^{-1}\ K^{-1})$ $\pm$ 10%
Pt	10.5	21400	134	2.87	30
PtSi	7.20	12378	201	2.49	18
Si in S1	70.0				114
Si in S2	81.4				133
Si in S3	82.4	2330	703	1.64	135
Si in S4	76.0				124
Si in S5	76.5				125

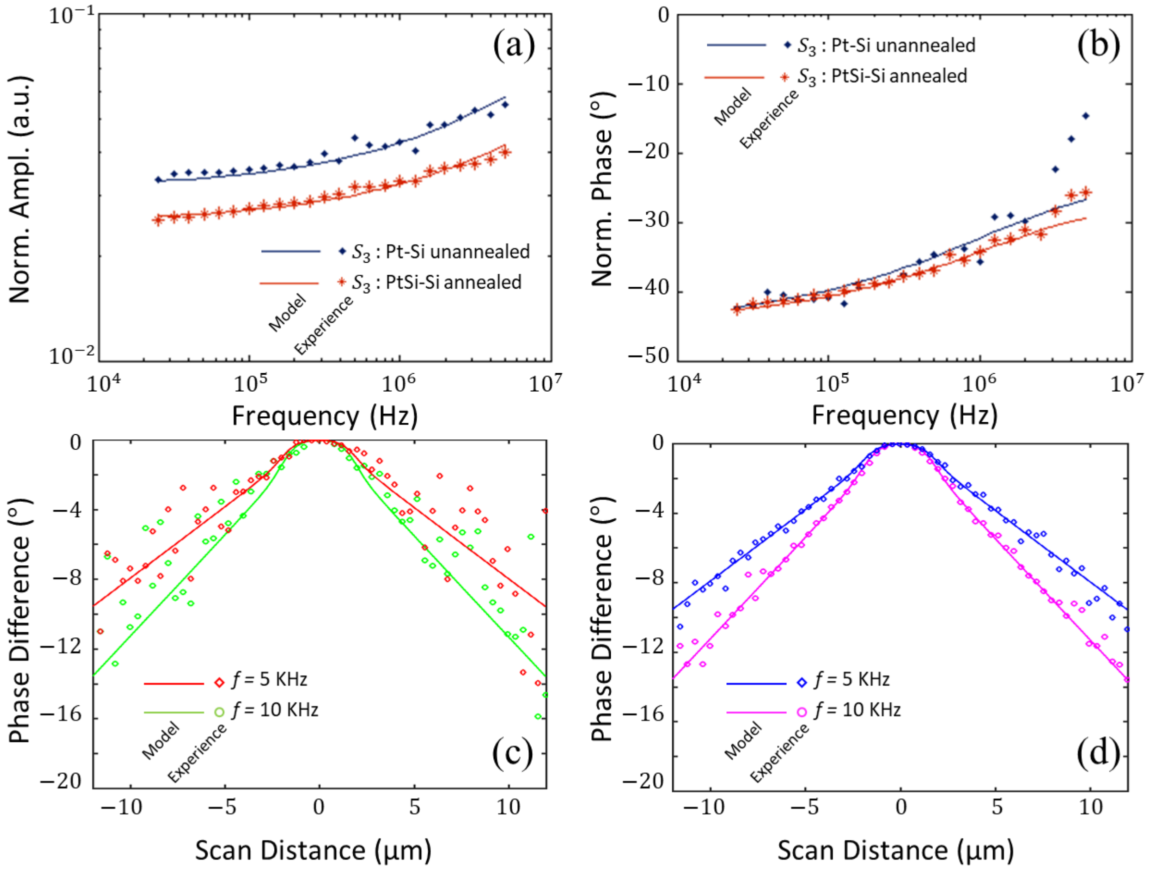

Figure S.4: Experimental and model fitting of (a) amplitude and (b) phase of surface temperature T_{AC} for the S3 intrinsic sample, using PTR; (c) and (d) represent the experimental and model for the unannealed and annealed S3 sample respectively, for two frequencies, using FSDTR.

As seen in Figure S.4(b), the phase of the annealed sample tends more to the limit of $-45°$ than the unannealed one. This implies that after annealing even if the thermal conductivity of the PtSi layer is lower, the heat is still better led to the substrate because of a lower TBR. The

normalized amplitudes (Fig. S.4 (a)) is not used to compare the two sample types because the emissivity has changed.

References

1. Costescu, R., Wall, M. & Cahill, D. Thermal conductance of epitaxial interfaces. *Phys. Rev. B* **67,** 54302 (2003).
2. Gundrum, B. C., Cahill, D. G. & Averback, R. S. Thermal conductance of metal-metal interfaces. *Phys. Rev. B* **72,** 245426 (2005).
3. Yang, J., Ziade, E. & Schmidt, A. J. Uncertainty analysis of thermoreflectance measurements. *Rev. Sci. Instrum.* **87,** (2016).
4. Y. Jarny, D. M. *Problèmes inverses et estimation de grandeurs en thermique, Métrologie thermique et techniques inverses*. (Cours C1A, Ecole d'Hiver METTI '99, Presses Universitaires de Perpignan, 1999).
5. Jcgm, J. C. F. G. I. M. Evaluation of measurement data — Guide to the expression of uncertainty in measurement. *Int. Organ. Stand. Geneva ISBN* **50,** 134 (2008).
6. Touloukian, Y. S. & Buyco, E. H. *Specific Heat nonmetallic solids Volume 5*. (IFI/Plenum, 1970).
7. Ye, N., Feser, J. P., Sadasivam, S. & Fisher, T. S. Thermal transport across metal silicide-silicon interfaces: An experimental comparison between epitaxial and nonepitaxial interfaces. *Phys. Rev. B* (2017).
8. Schubert, K. & Pfisterer, H. Zur Kristallchemie Der b-Metall-Reichsten Phasen in Legierungen Von Ubergangsmetallen Der Eisentriaden Und Platintriaden Mit Elementen Der Vierten Nebengruppe. *ZEITSCHRIFT FUR Met.* **41,** 433–441 (1950).
